# Onset of Oviposition Triggers Abrupt Reduction in Migratory Flight Behavior and Flight Muscle in the Female Beet Webworm, *Loxostege sticticalis*

**DOI:** 10.1371/journal.pone.0166859

**Published:** 2016-11-28

**Authors:** Yunxia Cheng, Lizhi Luo, Thomas W. Sappington, Xingfu Jiang, Lei Zhang, Andrei N. Frolov

**Affiliations:** 1 State Key Laboratory for Biology of Plant Diseases and Insect Pests, Institute of Plant Protection, Chinese Academy of Agricultural Sciences, Beijing, P. R. China; 2 USDA-Agricultural Research Service, Corn Insects & Crop Genetics Research Unit, Genetics Laboratory, Iowa State University, Ames, Iowa, United States of America; 3 All-Russian Institute for Plant Protection, Pushkin, Saint Petersburg, Russia; USDA Agricultural Research Service, UNITED STATES

## Abstract

Flight and reproduction are usually considered as two life history traits that compete for resources in a migratory insect. The beet webworm, *Loxostege sticticalis* L., manages the costs of migratory flight and reproduction through a trade-off in timing of these two life history traits, where migratory behavior occurs during the preoviposition period. To gain insight into how migratory flight and reproduction are coordinated in the female beet webworm, we conducted experiments beginning at the end of the preoviposition period. We used flight mills to test whether flight performance and supportive flight musculature and fuel are affected by the number of eggs oviposited, or by the age of mated and unmated females after onset of oviposition by the former. The results showed that flight distance, flight velocity, flight duration, and flight muscle mass decreased abruptly at the onset of oviposition, compared to that of virgin females of the same age which did not change over the next 7 d. These results indicate that onset of oviposition triggers a decrease in flight performance and capacity in female beet webworms, as a way of actively managing reallocation of resources away from migratory flight and into egg production. In addition to the abrupt switch, there was a gradual, linear decline in flight performance, flight muscle mass, and flight fuel relative to the number of eggs oviposited. The histolysis of flight muscle and decrease of triglyceride content indicate a progressive degradation in the ability of adults to perform additional migratory flights after onset of oviposition. Although the results show that substantial, albeit reduced, long-duration flights remain possible after oviposition begins, additional long-distance migratory flights probably are not launched after the initiation of oviposition.

## 1. Introduction

Long-distance flight and reproduction are energetically costly life history traits for migratory insects [1–4]. Costs engender trade-offs and hence covariation between life history traits (i.e., syndromes) [5], and optimal resource allocation to flight and reproduction is an important part of the life history strategy in migratory insect species. Resource trade-offs between flight-associated characters and reproduction are well-studied in wing-polymorphic insects [5–7]. Long-winged individuals have well developed flight muscles and produce fewer offspring, while flightless short-winged individuals have undeveloped flight muscles and produce more offspring. However, dispersal in wing-polymorphic species tends to be short-distance, and comparatively little detail is known about migration-reproduction trade-offs in long-range migrants, including many lepidopteran pests [4].

In highly disturbed environments, such as agricultural landscapes, both reproductive output and dispersal are expected to be favored in a colonizing insect species [7, 8]. This pattern is referred to as a colonizer syndrome [9, 10], and there are a number of case studies supporting this concept [11]. Nevertheless, given the significant energetic costs of both flight and reproduction, one also would expect intrinsic constraints on the extent to which increases in both can evolve, and there are many examples of species that do not exhibit the colonizer syndrome [11].

In many migratory insect species, migration is restricted to the preoviposition period. In some cases, such as the oriental armyworm, *Mythimna separata* (Walker) [12], oogenesis is suppressed until after migration, a pattern of physiological and behavioral covariation called the oogenesis-flight syndrome [1]. By limiting migratory flight to the preoviposition period, and reproduction to the post-migratory period, the insect can efficiently devote internal resources to both in sequence. Most research has focused on how resources are partitioned between flight and reproduction, and how certain reproductive resources may be affected by flight. Little is known about how this trade-off is further managed after reproduction is initiated following initial migration, or how further migration may be affected. In the cricket, *Velarifictorus parvus*, and the butterfly, *Pieris napi*, flight muscles degrade after migration, and the recovered proteins and lipids may support subsequent egg production [13, 14]. However, it is unclear if oviposition promotes the decline in flight capability or if the latter declines spontaneously at the end of migratory flight. Answering these questions is important for a complete understanding of the relationship between flight and reproduction as well as for modeling resource allocation in a migratory insect. Furthermore, in some species, such as the beet armyworm, *Spodoptera exigua* [15], migratory behavior and the oviposition period overlap. Thus, despite the substantial energetic costs involved, simultaneity of migration and reproduction is not automatically precluded physiologically or evolutionarily. The question that must be addressed separately for each migratory species is how the trade-offs are managed. Characterizing the relationship between the preoviposition period and migratory behavior is a critical first step.

The beet webworm, *Loxostege sticticalis* L. (Lepidoptera: Crambidae), is a destructive pest of crops and fodder plants grown in several areas of the northern temperate zone of North America, Eastern Europe and Asia, between latitudes 36°N and 55°N [16]. It is a facultative migrant species, with the decision to migrate or remain resident as an adult being made both in the larval and adult stage. It has multiple generations per year, and migration by spring, summer, and autumn generations has been studied by means of mark-release-recapture, radar, and molecular markers [17–19]. Beet webworm has a relatively long preoviposition period during which migratory behavior takes place, but the relationship between reproduction and migration is not simple for this species. When Kong et al. [20] reared larvae under moderate densities in the laboratory, the adult preoviposition period averaged about 7–9 d, compared to 5–6 d for females reared as isolated larvae. In that same study, flight duration, distance, and velocity of 1-d-old adults of mixed sexes were also highest when reared at moderate larval densities, and these flight parameters were positively correlated with preoviposition period. Optimal temperature for reproduction is 22–26°C, and the preoviposition period is longer at lower temperatures [21]. Migratory behavior may be extended when a recent immigrant encounters temperatures below 22°C [22]. Together, these studies suggest active coordination of the preoviposition period and migration in beet webworm [21]. However, the evidence for coordination remains inconclusive, because migratory flight seems to be undertaken primarily by moths aged 2–5 d, which have a preoviposition period of 5–7 d [23], and not by 1-d-old moths as were tested by Kong et al. [20]. Furthermore, length of the preoviposition period decreases linearly with increasing rearing temperature [21], consistent with the former simply reflecting temperature dependence of insect reproductive development (e.g., [24]). Thus, the observed long preoviposition period at low temperature is not necessarily an adaptation to support migration.

In coordinated radar and trapping studies in China, Feng et al. [17] observed beet webworms undertaking long-duration (~7 h) high-altitude migratory flight in the spring. About two-thirds of the migrating females were unmated with immature but developing ovaries, while the rest were mated with mature ovaries. Moths of later generations were also observed flying at high altitude, a typical signature of migratory flight [4], but these flights were of lesser, albeit substantial (~2–3 h), duration. Nevertheless, Jiang et al. [18] showed that adults that underwent larval diapause, as would be typical of those engaging in springtime migration, had prolonged preoviposition periods of ~10 d compared to 6 d in females reared from non-diapause larvae. Xie et al. [25] subsequently showed that long-distance flight performance of 3-d-old unmated adults (sexes pooled) on flight mills was positively influenced by diapause. Again the results are suggestive of an adaptive coordination of preoviposition period and migratory activity.

Cheng et al. [23] examined the effects of long-duration flight on reproduction. Such flight (averaging > 25 km/12 h on flight mills) by mated females at 2–5 d post-eclosion did not affect length of the preoviposition period, lifetime fecundity, mating frequency, or egg viability compared to unflown controls. It did narrow the temporal window of oviposition initiation, the period of first oviposition (PFO), among 3 and 5-d-old cohorts flown at the same age [23], indicating migratory flight and onset of oviposition are coordinated at some level. Reduced PFO improves oviposition synchrony, thus intensifying larval population outbreaks [23, 26], as long as adequate adult food resources are available [27].

Facultative migration in beet webworm most likely has evolved as a life-history strategy to cope with complex and variable environments in the temperate zone. We propose that migratory flights and reproduction are allocated to the preoviposition and post-migratory periods, respectively, to reduce resource competition between these energetically costly activities. Temporal segregation of migratory flight and oviposition is designed to contribute to reproductive success and successful colonization of ephemeral habitat. Under this scheme, continued migratory flight should be less necessary at, or after, the onset of oviposition, and one would expect a reduction in behavioral and physiological aspects of flight in the beet webworm with reallocation of flight-supporting resources to reproduction. This hypothesis is supported by the field observation that when adults begin oviposition, long-distance flight becomes less pronounced [17, 22].

Our main goal in this study was to better understand whether and how migratory flight and reproduction are coordinated in the female beet webworm. To gain insight into this question, we conducted experiments beginning at the end of the preoviposition period, i.e. at the onset of oviposition (5 to 7 d after emergence), to test whether subsequent flight performance and supportive flight musculature and fuel are affected by 1) the number of eggs oviposited, or 2) the age of mated and unmated females after onset of oviposition by the former. If reallocation of internal resources from migration to reproduction is actively managed in this species, we expected to see an abrupt switch in flight behavior, flight musculature, or flight fuel levels at the onset of oviposition compared to unmated (i.e., non-ovipositing) moths of the same age. Alternatively, if there is no substantial reallocation of resources, we expected to see no relationship of onset of oviposition to flight and supporting assets. If reallocation occurs but is indirect rather than specifically managed, we expected to see a linear decline in flight and supporting assets with increased numbers of eggs oviposited or over time as oviposition progresses.

## 2. Materials and Methods

### 2.1 Insect rearing

Beet webworms were collected from a field population in the Hebei Province of China (114.45°E, 41.73°N). In that area, no specific permissions were required for these types of field studies because no endangered or protected species were involved. The insects had reared in the laboratory for two generations before the experiment. Larvae were reared on lamb’s quarters, *Chenopodium album* L. (Chenopodiaceae), at 22°C, 75% relative humidity and 16:8 (L:D) photoperiod. They were kept at a density of 10 larvae per 650-ml jar, which is the optimal density to yield migrant adults [20]. After the eclosion of adults, one female and one male was randomly coupled in 245-ml cylinders constructed with transparent plexiglass. The chambers were lined with parchment paper on the inside wall and a piece of filter paper at the bottom. Adults were provided a 10% glucose solution via cotton wicks on the cage lid as food. Eggs were collected daily from the parchment paper, the filter paper and the lids. For the control groups, two same-age virgin females were put in a 245-ml cylinder at emergence and maintained as described above.

### 2.2 Experimental design

The influence of reproduction on flight performance, flight muscle dry mass and triglyceride content of mated female beet webworms was assessed by comparing them with virgins of the same age. The emergence date for each female was recorded, and each mated female was assigned a virgin counterpart of the same age as a control for comparison. Mated females were checked daily to determine the onset date of oviposition and daily fecundity. Age is expressed in groupings of time after onset of oviposition as mean age, and each time grouping has a standard error. Data on age (days after onset of oviposition) and realized fecundity (numbers of eggs laid) for mated individuals were collected on days 1, 3, 5, and 7 after onset of oviposition. Beet webworms oviposit most of their eggs during the first 7 d after onset of oviposition [23], so sample collection was limited to this period for both experiments in this study. In total, 403 pairs of mated and equivalent-aged virgin females were tested, including 124 pairs for determining the effects of age after onset of oviposition on flight performance, 111 pairs for determining the effects of number of eggs laid on flight performance, 84 pairs for determining the effects of age after onset of oviposition on triglyceride content, and additional 84 pairs for testing the effect of number of eggs laid on triglyceride content.

#### 2.2.1 Flight performance

Flight performance of female beet webworms was characterized using a 32-channel flight mill system [20]. Three parameters were examined: flight distance, flight velocity, and flight duration. Adults were attached by the dorsum of the first abdominal segment to the arm of the flight mill with 502 glue (Beijing Chemical Works, Beijing). Flight tests were performed in a dark room, maintained at 22°C and 70–75% relative humidity. Flight data of each female were recorded by the computer for 12 h from 19:00 through 07:00, a period optimal for studying beet webworm flight behavior [23].

#### 2.2.2 Flight muscle mass

After the flight test, the thorax and abdomen of each moth were dissected. The thorax was dried in an oven at 55°C for 15 h. Next, the dorsal longitudinal muscle was separated from the dorsal epidermis and the tergosternal muscles using dissecting needles. The dry mass of the longitudinal muscle was weighed on an electronic balance (XS205, Mettler Toledo, Switzerland) and used as a descriptor of flight muscle mass. Flight performance and the muscle mass data were omitted for any presumed-mated female in which no spermatophore was found, indicating it remained unmated.

#### 2.2.3 Triglyceride content

The females for the triglyceride content test did not experience the tethered flight. The triglycerides were isolated using a Tissue Triglyceride assay kit (Applygen Technologies, Beijing, China), following the manufacturer’s instructions. The head and wings were removed before the body was weighed and homogenized by pestle (Sangon Biotech, Beijing) in 1 ml of lysis solution in a 1.5-ml microcentrifuge tube. After centrifugation (12,000 rpm, 2 min) of the homogenate, the resulting supernatant was used to measure total triglyceride content on the basis of color developed from the glycerophosphate oxidase Trinder reaction, in which triglycerides are decomposed into glycerol and fatty acids. The glycerol is further transformed into quinoneimine dye, the optical density (OD) of which is proportional to triglyceride content. The OD was measured at a wavelength of 550 nm on an Infinite M200 spectrophotometer (Tecan) and then transformed into triglyceride concentration using a standard curve. The standard curve was generated from a 0.7-fold dilution series of 1.25μM glycerine over six concentrations. Triglyceride content per body weight (μg/mg) (C) was calculated with the equation: C = T / W, where T = total mass of triglycerides based on the OD value, and W = body weight (mg).

#### 2.2.4 Data analysis

The differences in flight distance, flight velocity, flight duration, dry flight muscle mass and triglyceride content between mated and virgin females were tested by paired-samples *t* test. Flight distance is essentially a product of the interaction between flight velocity and flight duration, so it is useful to examine it separately despite being correlated to some extent with both of the latter. For mated females, the influence of egg number on the above parameters was assessed by linear regression analysis. For virgin females, the influence of corresponding age grouping was measured with a Pearson’s correlation analysis. All the numeric values in this study are presented as the mean ± SE (standard error of mean). All statistical analyses were performed with SPSS software (SPSS 16.0)

## 3. Results

**Fig 1:**
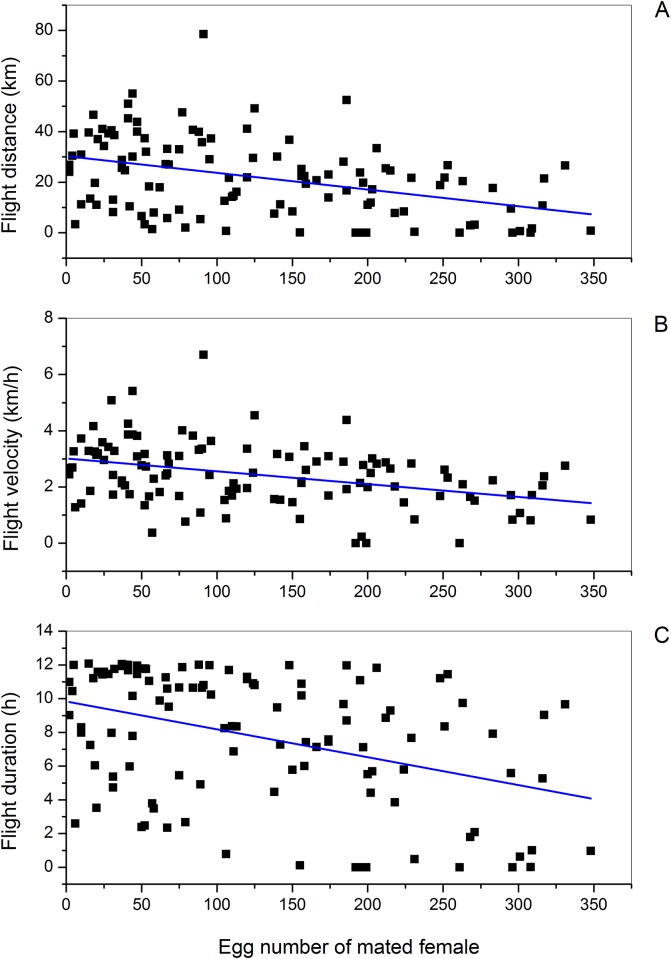
Influence of number of eggs oviposited on (A) flight distance, (B) flight velocity, and (C) flight duration of mated female beet webworms on flight mills. **(A)** Linear regression of flight parameters (Y) on the number of eggs laid (X) by mated females: *Y* = 30.30–0.066 *X*, *t* = -4.60, *P* < 0.0001, n = 111; **(B)** *Y* = 3.01–0.005 *X*, *t* = -4,24, *P* < 0.0001, n = 111; **(C)** *Y* = 9.84–0.02 *X*, *t* = -4.60, *P* < 0.0001, n = 111.

**Fig 2:**
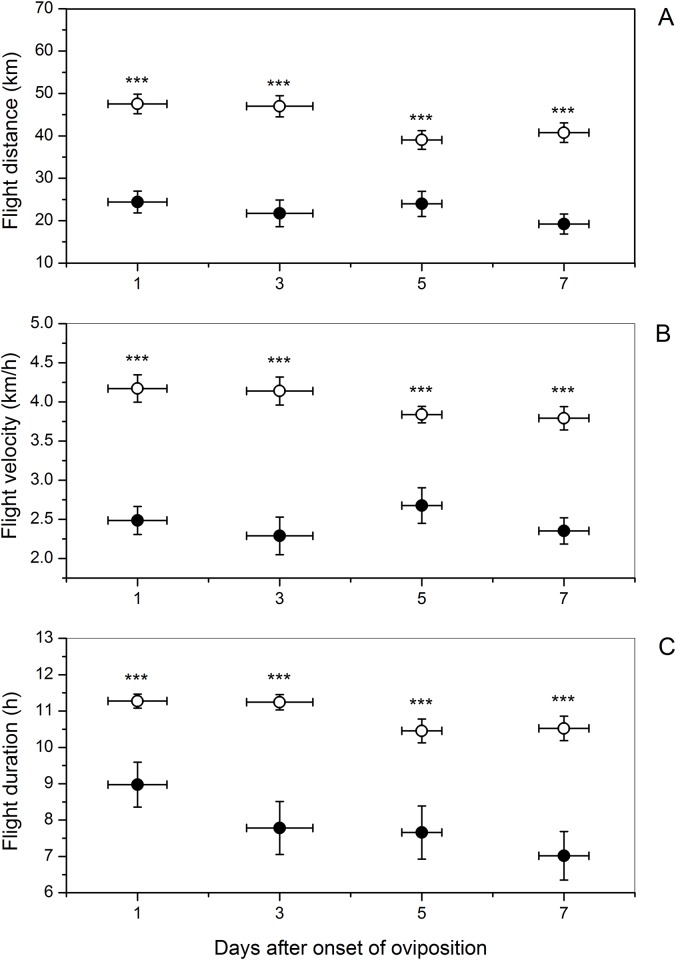
Influence of age groupings (days after onset of oviposition) on (A) flight distance, (B) flight velocity, and (C) flight duration in female beet webworms. Mean parameter values of mated (solid circle [●]) and virgin (open circle [○]) females of the same age. Vertical error bars indicate SE of mean parameter value, and horizontal error bars indicate SE of age within age grouping. Sample sizes of age-paired mated and virgin females from left to right are 32, 28, 30, and 34. Three asterisks (***) indicate a *P* value ≤ 0.001 analyzed by paired-samples *t* test.

Flight performance of mated female beet webworms decreased significantly in a linear fashion as the number of eggs oviposited increased from 2 to 348. Flight distance decreased from > 30 to < 10 km (Fig 1A; *Y* = 30.30–0.066 *X*, *t* = -4.60, *P* < 0.0001, n = 111), flight velocity decreased from 2.5 to 0.5 km/h (Fig 1B; *Y* = 3.01–0.005 *X*, *t* = -4,24, *P* < 0.0001, n = 111), and flight duration decreased from 10 to 2.5 h (Fig 1C; *Y* = 9.84–0.02 *X*, *t* = -4.60, *P* < 0.0001, n = 111) with increasing number of eggs oviposited. Flight distance and velocity of virgin females in the same age groupings as the mated females did not change significantly. However, flight duration of virgin females decreased with increasing age (Table 1). At each age grouping after onset of oviposition, flight distance, flight velocity, and flight duration of mated females were significantly and substantially less than among same-age virgin females. Across all age groupings after the beginning of egg laying, virgin females averaged 39 to 47 km of flight per night, whereas distance flown by mated females was roughly half, from 19 to 25 km (Fig 2A; all *t* ≥ 4.48, *P* < 0.0001). Flight velocity of virgin females averaged about 4 km/h, while that of mated females was only about 2.5 km/h across all age groupings (Fig 2B; all *t* ≥ 4.79, *P* < 0.0001). Flight duration of virgin females averaged from 10.45 to 11.27 h, whereas that of mated females was 7–9 h (Fig 2C; all *t* ≥ 3.53, *P* ≤ 0.001).

**Table 1:** Pearson’s correlations of flight distance (km), flight velocity (km/h), flight duration (h), dry flight muscle mass (mg/adult), and triglyceride content (mg/adult), with age grouping of virgin female beet webworms after onset of oviposition by age-paired mated females.

	*r*	n	*P*-value
**Flight distance**	**-0.23**	**111**	**0.05**
**Flight velocity**	**-0.07**	**111**	**0.49**
**Flight duration**	**-0.36**	**111**	**0.00**
**Flight muscle mass**	**0.12**	**111**	**0.08**
**Triglyceride content**	**-0.07**	**84**	**0.53**

**Fig 3:**
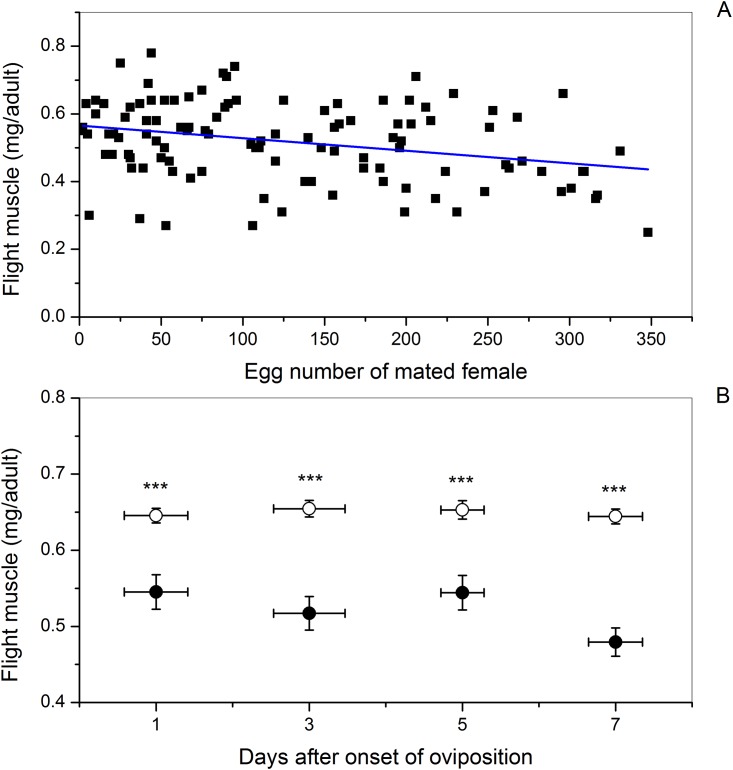
Influence of number of eggs oviposited (A) or age groupings (days after onset of oviposition) (B) on dry flight muscle mass of female beet webworms. **(A)** Linear regression of flight muscle mass (Y) on the number of eggs oviposited (X) by mated females: *Y* = 0.57–3.71E-4 *X*, *t* = 3.30, *P* = 0.001, n = 111. **(B)** Mean flight muscle mass in mated (circle [●]) and virgin (open circle [○]) females of the same age. Vertical error bars indicate SE of mean flight muscle mass, and horizontal error bars indicate SE of age within age grouping. Sample sizes of age-paired mated and virgin females from left to right are 32, 28, 30, and 34, respectively. Three asterisks (***) indicate a *P* value ≤ 0.001 analyzed by paired-samples *t* test.

The flight muscle mass of the mated females decreased linearly from approximately 0.55 to 0.45 mg/adult with increasing egg production (Fig 3A; *Y* = 0.57–3.71E-4 *X*, *t* = 3.30, *P* = 0.001, n = 111). Flight muscle mass in virgin females was not significantly correlated with the age groupings that corresponded to pairings with mated females (Table 1). Paired comparisons within all age groupings after onset of oviposition indicate significantly less flight muscle mass in mated females than in virgin females (Fig 3B; all *t* ≥ 3.81, *P* ≤ 0.001).

**Fig 4:**
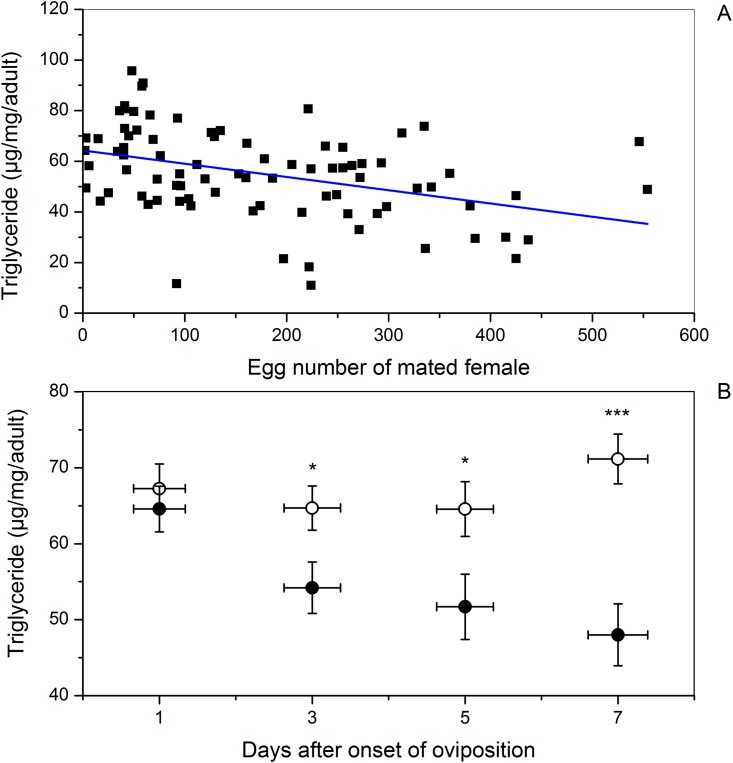
Influence of number of eggs oviposited (A) or age groupings (days after onset of oviposition) (B) on triglyceride content of female beet webworms. **(A)** Linear regression of triglyceride content (Y) on number of eggs oviposited (X) by mated females: *Y* = 64.27–0.05 *X*, *t* = -3.91, *P* < 0.0001, n = 84. **(B)** Mean triglyceride content in mated (circle [●]) and virgin (open circle [○]) females of the same age. Vertical error bars indicate SE of mean triglyceride content, and horizontal error bars indicate SE of age within age grouping. Sample sizes of age-paired mated and virgin females from left to right are 24, 21, 19, and 20. One asterisk (*) indicates a *P* value < 0.05, and three asterisks (***) indicates a *P* value ≤v0.001 analyzed by paired-samples *t* test.

The triglyceride content of mated females decreased linearly from 65 to 30 μg/mg/adult with increasing number of eggs ovipositied (Fig 4A; *Y* = 64.27–0.05 *X*, *t* = -3.91, *P* < 0.0001, n = 84), but it was not significantly correlated with the corresponding age groupings in virgin females (Table 1). At day 1 after onset of oviposition, triglyceride content in the mated females did not differ significantly from that of the virgins in the same age groupings (Fig 4B; *t* = 0.56, *P* = 0.58). However, on subsequent days after onset of oviposition, triglyceride content was significantly less in mated females than in corresponding virgin females (Fig 4B; all *t* ≥i2.15, *P* ≤ 0.04).

## 4. Discussion

At the end of the preoviposition period, there was an abrupt shift to decreased flight performance (duration, velocity, distance) and flight muscle mass compared to virgin females of the same age. Furthermore, measures of these parameters, except flight duration, in virgin females did not change significantly thereafter across corresponding ages. This points to an actively managed reallocation of resources away from migratory flight and into egg production triggered by the onset of oviposition, rather than a set of spontaneous behavioral and physiological adjustments associated with age itself. A negative correlation between flight duration and age in virgin females, may attribute to the fact that flight test was begun around 7 d after emergence, at which the peak migratory flight period had passed, and most of adults should have transferred to reproduction [23]. In addition to the abrupt switch in mated females, there was a gradual, linear reduction in flight performance, flight muscle mass, and flight fuel related to the number of eggs oviposited. At the rearing temperature used in this study, peak oviposition occurs on the second night after onset of ovipostion, and declines steadily with age thereafter [21], so the number of eggs oviposited in our study is probably related to age after onset of oviposition in the same way. The latter results indicate a progressive degradation in the ability of adults to perform additional migratory flights after onset of oviposition, in part via the breakdown of flight muscles and a reduction in energy reserves.

It is important to note that even though flight performance of beet webworm females was significantly reduced at the onset of oviposition, flight was by no means eliminated altogether. Considerable duration of flight was still possible and not uncommon among ovipositing females of all ages in our study. This is consistent with evidence that reproductively mature beet webworms that have begun oviposition, distribute their eggs within a relatively small radius (7–8 km) [22] compared to the 725–1117 km covered during long-distance migration [19]. Whether flight of somewhat long duration (e.g., 2–3 hours) after the onset of oviposition should be considered migratory flight is unclear. The substantial decrease (~40%) in flight velocity after onset of oviposition suggests flights by these females may not reflect migratory behavior. For example, in the black cutworm, *Agrotis ipsilon*, the velocity of short, trivial flights on flight mills was about half that of moths making long-duration migratory flights [28].

In the noctuid *Helicoverpa armigera*, mated females showed a 15-fold decrease in total flight duration and a 28-fold decrease in duration of the longest continuous flight compared with unmated females of the same age [29]. In that study, unmated females also oviposited, presumably because oocytes continue to develop and exceed available space in the ovaries unless mature eggs are expelled. The effects on flight were therefore triggered by mating, not by oviposition itself. For beet webworms, the abrupt change in behavior after onset of oviposition and lack of subsequent change in unmated moths as they age suggest that oviposition of fertilized eggs triggers the response. Another long-distance migratory insect, *M*. *separata*, makes multi-generation stepwise migrations between southern and northern regions of China each year. Flight performance of this species increases with adult age before oviposition and decreases after the onset of oviposition. Flight propensity was essentially eliminated after the adults had oviposited for 6 d or when > 1,200 eggs were produced [30].

In the beet webworm, the decrease in flight performance at the end of the preoviposition period represents a timing trade-off to manage the energetic costs of flight and reproduction. Migration promotes synchrony of reproduction in immigrant populations of this species by tightening the window of onset of oviposition, resulting in temporal concentration of larvae [23]. Reduction in additional long-distance movement once oviposition starts, as revealed by the data in the current study, presumably promotes spatial concentration of immigrant offspring in the new habitat. Together, these strategies may lead to high density larval populations, which in an agricultural setting is experienced as a pest outbreak.

A number of migratory insect species exhibit different life-history strategies than those of the beet webworm and others that temporally segregate migration and oviposition. For instance, adults of the beet armyworm, *Spodoptera exigua*, can mate on the night of emergence and oviposit the following night. The migratory flights of this species are synchronous with oviposition, and it seems that flight capacity is not adversely affected even after 7 d of oviposition [15]. This mirrors strategies of certain insects that do not engage in long-distance migratory flights at all. Flight performance of adult *Adelphocoris* spp. was greatly affected by age but not by gender, mating status, or oviposition [31]. Mated females of *Cydia pomonella* exhibited peak flight performance between 1 and 3 d after eclosion, corresponding to the major egg-laying period [32]. Therefore, different effects of reproduction on flight performance are due to differences in life-history strategies across insect species.

Resources liberated by histolysis of flight muscles at the end of the preoviposition period in beet webworm are probably reallocated to reproduction. For instance, in a cricket, *Modicogryllus confirmatus*, under food-limited conditions, dealate long-winged females produced more eggs than short-winged females because the former could use the nutrients derived from the flight muscle histolysis for egg production [33]. Although flight muscle histolysis in another cricket, *Gryllus bimaculatus*, can release nutrients for use elsewhere, they respresent only a small proportion of the total demand for oogenesis [34]. On the other hand, maintenance alone of flight muscle is costly [34], and histolysis of flight muscles to save maintenance costs represents an indirect pathway providing additional resources for reproduction during the post-migratory stage. This pathway of resource reallocation may be common in other species which temporally segregate migration and reproduction as part of their life-history strategy. The breakdown of flight muscle in beet webworm suggests lack of need for migratory flight and the muscles supporting it after the initiation of oviposition. In addition, or alternatively, the histolysis of flight muscle may directly diminish the probability of launching further long-distance migration.

Factors triggering breakdown of flight muscles after migratory flight differ among species. Johnson [35] found that the flight muscles began to break down a few days after alate aphids settled on their host plants. Onset of flight muscle degeneration in alate aphids was delayed by preventing settling, either by denying access to the host or by leaving them on a poor host in a dark room for several days. Flight muscle histolysis in female *Dysdercus intermedius* was controlled by both food and mating. Starvation inhibits flight muscle histolysis and production of eggs. Copulation stimulates histolysis both in the non-starved and starved females [36]. Age is an important factor in the breakdown of flight muscle in crickets, in which flight muscle mass increased significantly to a maximum at day 2 and 3 after the final moult and continued at a high rate until day 10. Flight performance decreased and the flight muscles started to histolyse after day 10 with the onset of ovarial growth [34]. These examples correspond to species-dependent life-history strategies. When the migratory flight is synchronous with reproduction, oviposition may have little impact on flight muscle. Alternatively, if migration is limited to the preoviposition period, onset of oviposition can accelerate flight muscle breakdown during the post-migratory stage, as we found for beet webworm.

Triglyceride is a major fuel source for supporting long-range flight in the beet webworm [37]. Triglyceride concentration decreased linearly with increased egg production in mated females. However, it did not decrease until day 3 of oviposition compared with same-age virgin females. This pattern differs from those of flight performance and flight muscle mass, which decreased to a new level by day 1 of oviposition. This result indicates that progressive oviposition itself is the main factor that causes the progressive decline in triglyceride, rather than active management triggered abruptly by the onset of oviposition. The net decrease in triglyceride in ovipositing females may result from their transformation to phospholipids which are internalized by developing eggs [6, 38–40] with the help of a lipophorin protein [41]. In beet armyworm, adults can burn triglycerides at a rate of 0.07 μg/sec during flight [42]. In the current study, total triglyceride reserves declined by more than 30% by day 7 of oviposition in mated female beet webworms. Thus, triglyceride reserves in ovipositing females may not be sufficient to allow further long-duration migratory flights.

Migration is an energetically costly activity that risks encountering worse environments, new enemies, and other hazards [7] in searching for a suitable habitat for offspring. However, onset of oviposition may be a signal that the purpose of migration is completed, and that additional migratory flight is unnecessary or would be counterproductive given the imperative to reproduce in the short time remaining before death. The breakdown of flight muscles and the reduction of triglyceride content degrade flight performance structurally and energetically, reducing the ability of female beet webworms to undertake additional long-distance migratory flight after the initiation of oviposition. This inference is supported by a field study, in which adult beet webworms remained within 7–8 km of the initial oviposition site [22], and were unlikely to launch longer distance flights during this life stage. By sequentially segregating migratory flight and oviposition activities, adult beet webworms manage the costs of resource competition between them. The resource allocation and acquisition pathways that have evolved in beet webworm appear to optimize the utilization of resources for migration during the preoviposition period, as it does for oviposition during the post-migratory stage. This scheme is a variation of the resource allocation strategies of species with differentiated morphs (short-/long-winged, alate/apteral, migrant/resident), and may apply to other lepidopteran pests that migrate only during the preoviposition period.

## Supporting Information

S1 Excel Data of all the figures and tables. (XLSX)
